# Progressive Inter-Path Interference Cancellation Algorithm for Channel Estimation Using Orthogonal Time–Frequency Space

**DOI:** 10.3390/s24134414

**Published:** 2024-07-08

**Authors:** Mauro Marchese, Henk Wymeersch, Paolo Spallaccini, Pietro Savazzi

**Affiliations:** 1 Department of Electrical, Computer and Biomedical Engineering, University of Pavia, 27100 Pavia, Italy; mauro.marchese01@universitadipavia.it; 2 Department of Electrical Engineering, Chalmers University of Technology, 412 96 Gothenburg, Sweden; henkw@chalmers.se; 3 HCL Software, Vimodrone, 20055 Milan, Italy; paolo.spallaccini@hcl.software; 4 Department of Electrical, Computer and Biomedical Engineering, University of Pavia and CNIT Consorzio Nazionale Interuniversitario per le Telecomunicazioni—Unità di Pavia, 27100 Pavia, Italy

**Keywords:** OTFS, channel estimation, (DD) delay-Doppler domain, fractional parameter estimation, inter-path interference

## Abstract

Fractional delay-Doppler (DD) channel estimation in orthogonal time–frequency space (OTFS) systems poses a significant challenge considering the severe effects of inter-path interference (IPI). To this end, several algorithms have been extensively explored in the literature for accurate low-complexity channel estimation in both integer and fractional DD scenarios. In this work, we develop a variant of the state-of-the-art delay-Doppler inter-path interference cancellation (DDIPIC) algorithm that progressively cancels the IPI as estimates are obtained. The key advantage of the proposed approach is that it requires only a final refinement procedure reducing the complexity of the algorithm. Specifically, the time difference in latency between the proposed approach and the DDIPIC algorithm is almost proportional to the square of the number of estimated paths. Numerical results show that the proposed algorithm outperforms the other channel estimation schemes achieving lower normalized mean square error (NMSE) and bit error rate (BER).

## 1. Introduction

Reliable communication in doubly dispersive channels, where high delay and Doppler spreads due to multipath and mobility are experienced, is becoming an increasingly prominent research topic toward the development of next-generation wireless networks [1,2]. The widely adopted OFDM (orthogonal frequency division multiplexing) is suitable for static frequency-selective multipath channels but experiences drastic performance degradation in high-mobility scenarios due to the loss of orthogonality between the subcarriers, as analyzed in [3], inducing intercarrier interference (ICI). To cater to high-mobility scenarios, several novel waveforms have been introduced in literature. An overview of some of the existing modulation schemes for high-mobility wireless channels is reported in [4]. Orthogonal time–frequency space (OTFS) modulation was proposed in [5,6] and is garnering a lot of attention due to its robustness against the Doppler effect. OTFS is a two-dimensional modulation scheme that multiplexes information symbols in the delay-Doppler domain, where the channel representation becomes sparse and the channel can be considered almost time-invariant [5,6,7]. In contrast, OFDM multiplexes information symbols in the time–frequency (TF) domain, where mobility generates a frequency response of the time-varying channel.

### 1.1. Problem Context

In this work, we consider a scenario comprising a single-antenna transmitter and receiver, focusing on the channel estimation problem with fractional delays and Doppler shifts and no prior information about the number of propagation paths [8,9]. The transmitter sends a pilot symbol in the delay-Doppler (DD) domain, while the receiver processes the received pilot frame to extrapolate the knowledge of the channel parameters.

The channel estimation problem is often considered in the *integer DD scenario,* which is facilitated by the assumption of having a channel with delays and Doppler shifts that are multiples of delay and Doppler resolutions. In this case, there is no interference between adjacent DD bins, and a simple threshold method can be used to obtain good performances, as shown in [10]. On the other hand, in reality, this assumption may not hold, which motivates the study of *fractional DD scenario,* which is more challenging, since the received replica of the pilot symbol in the DD domain spreads into adjacent bins after 2D sampling of the DD domain [7,11]. This is the well-known inter-path interference phenomenon. Our focus is on the effects of IPI caused by fractional DDs and IPI cancellation to recover channel state information (CSI) with high accuracy.

### 1.2. State of the Art

Channel estimation in OTFS systems has been widely investigated in the literature since the detection performance of the OTFS receiver is based on the estimated channel matrix [12,13,14,15]. In [10], an embedded-pilot-aided method for channel estimation is proposed, achieving good performances in both integer DDs and fractional Doppler scenarios. Channel estimation in massive multiple-input multiple-output scenarios (MIMO) has been investigated in [16], where additional sparsity due to a large number of transmit and receive antennas allows the channel estimation problem to become a simpler sparse recovery problem taking advantage of the angular domain. In [17,18,19] a novel tensor-based approach for channel estimation in massive MIMO systems is proposed to further exploit channel sparsity in the delay-Doppler-angle domain and reduce signal processing burden. Low-complexity channel estimation schemes were considered in single-antenna systems in [8], where modified maximum likelihood estimator (M-MLE) and two-step estimator (TSE) algorithms were introduced. The first is based on the asymptotic orthogonality of a delay-Doppler parameter matrix for large OTFS frames, which allows for the simplification of the cost function. On the other hand, the second one has lower complexity and relies on disjoint delay and Doppler estimation. Under the assumption of having good separability of the received pilot replica due to fine delay and Doppler resolutions, in both algorithms, the IPI is cancelled, and at each iteration, a residual received vector is used for estimating channel parameters of the next path. Similarly, IPI cancellation is performed in the low-complexity channel estimation scheme for discrete Zak transform-based OTFS (DZT-OTFS) proposed in [20]. Sparse Bayesian learning (SBL) algorithms for channel estimation are also considered in [21,22], but M-MLE and TSE algorithms are shown to perform better then SBL in [8] when delay and Doppler resolutions are fine enough. In [23], different channel estimation schemes for the fractional DD case are proposed with the scope of improving the spectrum efficiency and reduce the peak-to-average-power-ratio (PAPR) by sending pilots in the time–frequency (TF) domain. The problem of high-precision channel estimation in IPI scenarios has been addressed in [11], where an iterative delay-Doppler IPI cancellation (DDIPIC) algorithm was proposed. In this case, the IPI cancellation is performed indirectly through refinement procedures carried out at each iteration. These refinements of the channel parameters take time, increasing the computational burdening of the channel estimation algorithm. In more detail, the computational complexity of the cost function, which is evaluated multiple times during each iteration, increases with the cube of the iteration index due to matrix inversion. However, in [11], the DDIPIC algorithm is shown to perform better than the M-MLE algorithm. Hence, in this work, we considered only the comparison between the proposed approach and the state-of-the-art DDIPIC algorithm.

### 1.3. Contributions

The goal of this work is to propose a variant of the DDIPIC algorithm from [11], named the progressive IPI cancellation (P-IPIC) algorithm, which cancels the IPI by performing residue cost function (RCF) maximization at each step. The main difference of the proposed algorithm with respect to the DDIPIC is that it operates in two phases: A *search phase*, where a certain number of possible paths are detected and a first rough estimate of the channel parameters is provided, A *refinement phase*, where the found paths are refined, looking for ones declared as false alarms and discarded, until the stopping criterion is met. This procedure allows the correct number of paths to be found while the accuracy of the estimates is improved. The contributions of this work can be summarized as follows:

- *Progressive IPI cancellation*: Instead of discovering possible paths and performing refinements in each iteration maximizing the observation-based cost function as in [11], we perform maximization of a residue-based cost function (RCF) at each step during the discovery phase. This progressive cancellation of the IPI allows a first channel estimate to be provided, aiming to reduce the residue energy as iteration progress, achieving high accuracy in channel matrix reconstruction.
- *Latency reduction due to single global refinement of the estimates*: The main drawback of the state-of-the-art DDIPIC algorithm is that it performs refinement procedures at each iteration, as discussed in Section 3.5. Progressive IPI cancellation allows the latency to be reduced, since, as discussed above, the algorithm can operate in two distinct phases and a single global refinement is needed.
- *Computational complexity reduction in search phase*: During the iterative search phase, the proposed algorithm looks for new paths by performing a RCF maximization. This allows us to reduce the computational cost since the computation of the RCF does not require matrix inversion. Moreover, the computational cost does not depend on the iteration index. As a consequence, coarse and fine estimation procedures described in Section 3.4 will take less time.
- *Resilience towards high-IPI scenarios*: In the literature, it is commonly assumed to have sufficiently fine delay and Doppler resolutions such that all the propagation paths are resolvable in the DD domain. In some cases, this may not hold, and robust low-complexity channel estimation algorithms must be designed to suit high-IPI scenarios. In this work, we consider both low- and high-IPI regimes, showing that the proposed approach is suitable for both regimes.

The remainder of this paper is organized as follows. In Section 2, the OTFS signal model is presented. In Section 3, OTFS channel estimation is covered, including the baseline DDIPIC method and our proposed P-IPIC method.

## 2. OTFS Modulation

In this section, the employed models for the OTFS signal, the propagation channel, and receiver-side processing are presented.

### 2.1. Transmitted Signal Model

In OTFS systems [5,7], the TX arranges MN symbols in the delay-Doppler grid, referred to as $I_{DD}$, where *M* is the number of subcarriers and *N* is the number of time slots of the OTFS frame. The DD-domain symbols are then converted to the time–frequency domain through inverse symplectic finite Fourier transform (ISFFT). Denoting with $X\in C^{M\times N}$ the delay-Doppler symbols multiplexed in the DD domain, the corresponding symbols in the time–frequency grid are obtained as

(1) $$X_{tf}\left[l,k\right]=\frac{1}{\sqrt{MN}}\sum_{n=0}^{N-1}\sum_{m=0}^{M-1}X\left[m,n\right]e^{j2\pi \left(\frac{nk}{N}-\frac{ml}{M}\right)}.$$

After that, the time-domain OTFS signal is obtained through the the Heisenberg transform as

(2) $$s\left(t\right)=\sum_{k=0}^{N-1}\sum_{l=0}^{M-1}X_{tf}\left[l,k\right]g_{tx}\left(t-kT\right)e^{j2\pi l\Delta f(t-kT)},$$

where $g_{tx}\left(t\right)$ is the transmit-shaping pulse of duration *T*. The continuous-time signal in Equation (2) has a total duration of NT and a bandwidth of MΔf, where *T* corresponds to the time slot duration and Δf=1/T is the subcarrier spacing. Thus, the delay and Doppler resolutions are, respectively,

(3) $$\tau_{\mathrm{res}}=\frac{1}{M\Delta f},\;\nu_{\mathrm{res}}=\frac{1}{NT}.$$

### 2.2. Channel Model

The transmitted signal passes through the channel that in the DD domain is modeled as

(4) $$h\left(\tau ,\nu \right)=\sum_{i=1}^{P}g_{i}\delta \left(\tau -\tau_{i}\right)\delta \left(\nu -\nu_{i}\right).$$

where *P* is the number of propagation paths and $g_{i}$, $\tau_{i}$, and $\nu_{i}$ are the complex channel gain, the propagation delay, and the Doppler shift of the *i*-th path, respectively.

### 2.3. Receiver Processing

The received signal is obtained by computing the Heisenberg transform and adding additive white Gaussian noise (AWGN) with monolateral power spectral density (PSD) $N_{0}$, referred to as n(t). Thus, the received signal is

(5) $$r\left(t\right)=\;\int \int \;h\left(\tau ,\nu \right)s\left(t-\tau \right)e^{j2\pi \nu (t-\tau )}d\tau d\nu +n\left(t\right),$$

At the receiver side, inverse operations are performed to recover the received symbols in the DD domain. Firstly, the receiver computes the Wigner transform by sampling the so-called cross-ambiguity function (CAF) $A_{g_{rx},r}\left(f,t\right)$ to convert the received time-domain signal into samples in the time–frequency grid as

(6) $$Y_{tf}\left[l,k\right]=A_{g_{rx},r}{\left(f,t\right)|}_{f=l\Delta f,t=kT}=\int r\left(t^{\prime }\right)g_{rx}^{*}\left(t^{\prime }-t\right)e^{-j2\pi f(t^{\prime }-t)}dt^{\prime }{|}_{f=l\Delta f,t=kT}.$$

Secondly, a SFFT is applied to obtain the DD-domain received frame

(7) $$Y\left[m,n\right]=\frac{1}{\sqrt{MN}}\sum_{k=0}^{N-1}\sum_{l=0}^{M-1}Y_{tf}\left[l,k\right]e^{-j2\pi \left(\frac{nk}{N}-\frac{ml}{M}\right)}.$$

From now on, we are going to consider OTFS modulation with rectangular shaping filters of duration *T* and amplitude $1/\sqrt{T}$ at both the transmitter and receiver side.

## 3. OTFS Channel Estimation

The purpose of channel estimation is to determine the parameters of (4), based on pilot signals. In this section, the pilot model and corresponding observation model are presented, followed by the baseline method and the proposed method.

### 3.1. Pilot Model

In our scenario [8,11], the single-antenna transmitter sends a pilot symbol in the DD domain. The corresponding pilot frame is given by

(8) $$X\left[m,n\right]=\left\{\begin{array}{cc} \sqrt{E_{p}} & m=m_{p},\;n=n_{p},\\ 0 & \mathrm{otherwise}, \end{array}\right.$$

where $m=m_{p}$, $n=n_{p}$ is the delay-Doppler resource element (DDRE), in which the pilot is sent and $E_{p}$ is the energy of the transmitted pilot signal.

At the receiver, after the operations described above and vectorization, the received pilot frame is given by

(9) y=vec(Y)=Hvec(X)+w=Hx+w,

where $w∼\mathrm{CN}\left(0,N_{0}I\right)\in C^{MN\times 1}$ is the AWGN noise vector and $H\in C^{MN\times MN}$ is the delay-Doppler domain channel matrix, given by

(10) $$H=\sum_{i=1}^{P}g_{i}G_{i}\left(\tau_{i},\nu_{i}\right),$$

where $G_{i}\left(\tau_{i},\nu_{i}\right)\in C^{MN\times MN}$ is a matrix that captures the effect of the propagation delay and the Doppler shift of the *i*-th path, and for $l^{\prime },l^{\prime \prime }=0,1,..,M-1$, $k^{\prime },k^{\prime \prime }=0,1,...,N-1$ is computed as [8,11]

(11) $$G_{i}\left[k^{\prime }M+l^{\prime },k^{\prime \prime }M+l^{\prime \prime }\right]=\frac{e^{-j2\pi \tau_{i}\nu_{i}}}{MN}\sum_{n=0}^{N-1}\sum_{m=0}^{M-1}r_{k^{\prime \prime },l^{\prime }}\left(m\right)e^{j2\pi \left(\frac{m}{M}\left(l^{\prime }-l^{\prime \prime }-\frac{M\tau_{i}}{T}\right)-\frac{n}{N}\left(k^{\prime }-k^{\prime \prime }-\frac{N\nu_{i}}{\Delta f}\right)\right)},$$

where

(12) $$\begin{array}{c} r_{k^{\prime \prime },l^{\prime }}\left(m\right)=\sum_{s=-m}^{M-1-m}e^{j2\pi s\frac{l^{\prime }}{M}}[\left(1-\frac{\tau_{i}}{T}\right)e^{j\pi \left(1+\frac{\tau_{i}}{T}\right)\left(\frac{\nu_{i}}{\Delta f}-s\right)}sinc\left(\left(1-\frac{\tau_{i}}{T}\right)\left(\frac{\nu_{i}}{\Delta f}-s\right)\right)+\\ \frac{\tau_{i}}{T}e^{-j2\pi \frac{k^{\prime \prime }}{N}}e^{j\pi \left(\frac{\tau_{i}}{T}\right)\left(\frac{\nu_{i}}{\Delta f}-s\right)}sinc\left(\tau_{i}\left(\nu_{i}-s\Delta f\right)\right)]. \end{array}$$

If the channel coefficients are normalized such that $\sum_{i=1}^{P}{\left|g_{i}\right|}^{2}=1$, the received pilot signal power equals the transmitted one. Under this hypothesis, the pilot signal-to-noise ratio (PSNR) can be computed as in [8]. Hence, the pilot signal power is the total energy $E_{p}$ divided by the overall time duration NT, while the noise power is the product of the power spectral density $N_{0}$ and the bandwidth MΔf. Thus,

(13) $$\mathrm{PSNR}=\frac{E_{p}/NT}{M\Delta fN_{0}}=\frac{E_{p}}{MNN_{0}}.$$

During a channel coherence window, the receiver processes the received pilot frame running a channel estimation algorithm, and once the estimated channel matrix $\hat{H}$ is obtained, uses the estimate to detect the incoming data frames.

### 3.2. Observation Model for Channel Estimation

As in [11], channel estimation is performed considering the input–output relation in Equation (9), rewritten as

(14) $$y=R\left(\tau ,\nu \right)g+w,$$

where $g={\left[g_{1}\;g_{2}\;...\;g_{P}\right]}^{T}\in C^{P\times 1}$ is the channel gains vector and $R\left(\tau ,\nu \right)=\left[r_{1}\;r_{2}\;...\;r_{P}\right]\in C^{MN\times P}$ is a matrix called constituent delay-Doppler parameter matrix (CDDPM). Each column of $R\left(\tau ,\nu \right)$ is related to a different path and is given by

(15) $$r_{i}\left(\tau_{i},\nu_{i}\right)=G_{i}\left(\tau_{i},\nu_{i}\right)x.$$

Given the input–output relation in Equation (14), the maximum likelihood (ML) estimate of the unknowns is given by

(16) $$\left(\hat{g},\hat{\tau },\hat{\nu }\right)=\arg\min_{g,\tau ,\nu }{∥y-R\left(\tau ,\nu \right)g∥}^{2}.$$

Although the minimization problem depends on three parameters, it can be simplified performing a two-step ML estimate of the unknowns as

(17) $$\left(\hat{\tau },\hat{\nu }\right)=\arg\max_{\tau ,\nu }\Phi \left(R\left(\tau ,\nu \right),y\right),$$

and

(18) $$\hat{g}={\left(R^{H}\left(\hat{\tau },\hat{\nu }\right)R\left(\hat{\tau },\hat{\nu }\right)\right)}^{-1}R^{H}\left(\hat{\tau },\hat{\nu }\right)y.$$

Here, the cost function $\Phi \left(R\left(\tau ,\nu \right),y\right)$ is computed as

(19) $$\Phi \left(R\left(\tau ,\nu \right),y\right)=y^{H}R\left(\tau ,\nu \right){\left(R^{H}\left(\tau ,\nu \right)R\left(\tau ,\nu \right)\right)}^{-1}R^{H}\left(\tau ,\nu \right)y.$$

The DDIPIC algorithm proposed in [11] performs an iterative maximization of the cost function in Equation (17) in order to jointly estimate delays and Dopplers of the paths. The operation of the algorithm is summarized below.

### 3.3. DDIPIC Algorithm

The algorithm proceeds iteratively for a maximum of $P_{\max}$ iterations and the CDDPM matrix is initialized as $R\left(\tau ,\nu \right)=0$. The details of the DDIPIC algorithm are provided in Algorithm 1. At each iteration, the parameters of one path are estimated as follows:

1. *Coarse estimation (line 2)*: The cost function in (19) is maximized over the DD grid of integer multiples of delay and Doppler resolutions, providing a coarse estimate of delay and Doppler shift. The search area is limited to $\left[0,L\tau_{\mathrm{res}}\right]\times \left[-K\nu_{\mathrm{res}},K\nu_{\mathrm{res}}\right]$, where $L=\lceil \frac{\tau_{\max}}{\tau_{\mathrm{res}}}\rceil$ and $K=\lceil \frac{\nu_{\max}}{\nu_{\mathrm{res}}}\rceil$.
2. *Fine estimation (lines 5–11)*: An iterative procedure runs for a maximum of $s_{max}$ iterations to provide a better estimate of the off-grid DDs. In each iteration, the cost function in (19) is maximized over a search area comprising fractional delays and Dopplers around, for s=1, the coarse estimate obtained in step 1 or, for s>1, the fine estimate obtained in the previous iteration. The search space is narrowed down as the iteration progresses, reducing the delay and Doppler search widths (lines 6–7). The fine estimation procedure ends when the stopping criteria for τ and ν are met. The search space for the fine estimation procedure is called $F_{DD}^{\left(s\right)}$, and we define $\Gamma =\{-\left\lfloor \frac{m_{\tau }}{2}\right\rfloor ,...,0,...,\left\lfloor \frac{m_{\tau }}{2}\right\rfloor \}$ and $\Lambda =\{-\left\lfloor \frac{n_{\nu }}{2}\right\rfloor ,...,0,...,\left\lfloor \frac{n_{\nu }}{2}\right\rfloor \}$ where $m_{\tau }$ and $n_{\nu }$ are design parameters. Notice that after the Cartesian product (denoted ×) of the two sets, only positive delays need to be considered. The parameters $\epsilon_{\tau }$ and $\epsilon_{\nu }$ are chosen to achieve the desired accuracy compatibly with $s_{\max}$.
3. *Check stopping criterion (line 15–19)*: A residue vector is computed as $E^{\left(i\right)}=y-R\left(\hat{\tau },\hat{\nu }\right)\hat{g}$ and if $∥E^{\left(i\right)}∥<\epsilon$ is satisfied, the algorithm stops and a number of $\hat{P}$ paths are detected; otherwise, before starting the search for a new path, all previous estimates are refined as described below. The convergence tolerance parameter ϵ for the stopping criterion must be chosen not to underestimate the number of propagation paths, not to miss those with smaller amplitudes, and not to overestimate the number of propagation paths due to residual IPI or noise. Since the noise is Gaussian, a reasonable value could be set taking into account the fact that the number of symbols per frame is MN, and the noise variance is $N_{0}$, so that we have set its value to $\epsilon =3\sqrt{MNN_{0}}$.
4. *Refinement procedure (line 20–21)*: All coarse and fine estimates are performed again for all the previously detected paths until the current one. In particular, at the *i*-th iteration, lines 2–13 are run again iteratively for $i_{\mathrm{ref}}=1,...,i$.

**Algorithm 1:** DDIPIC
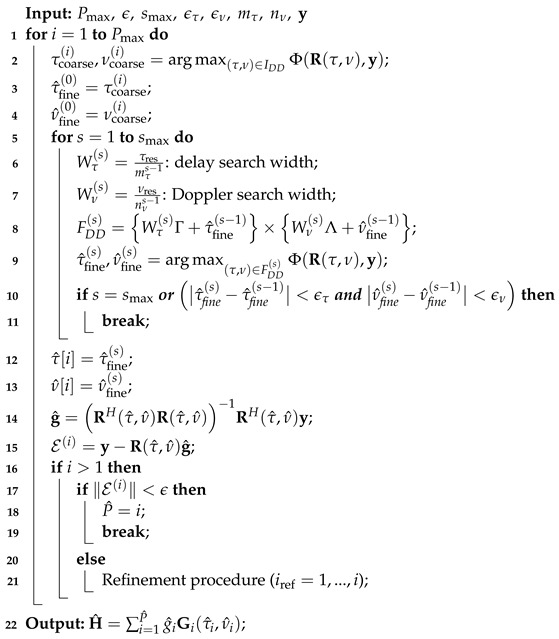


### 3.4. Proposed P-IPIC Algorithm

The proposed variant of the DDIPIC algorithm aims to reduce the complexity of DDIPIC and is based on a RCF maximization and performs an iterative search as in the original algorithm until the maximum number of iterations is reached or the stopping criterion is met. The pseudocode of the proposed P-IPIC algorithm is provided in Algorithm 2. The main parameters are as for the DDIPIC algorithm.
**Algorithm 2:** Proposed P-IPIC
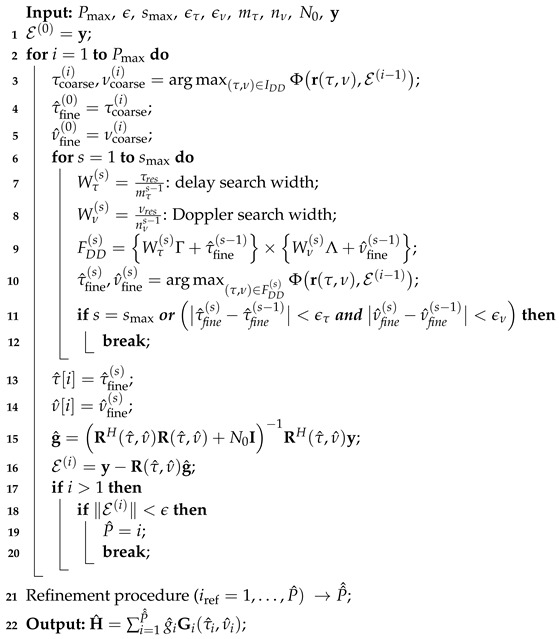


The proposed algorithm features several key differences compared to Algorithm 1:

- *Coarse and fine estimation based on the residuals (lines 3 and 10):* The key idea is that once a path is detected and the residue vector is obtained, during the next search, a new path is discovered looking at the residual pilot signal of the previous iteration. Thus, at the *i*-th iteration, the algorithm looks for a new path maximizing an RCF given by
  (20) $$\begin{array}{cc} \Phi \left(r\left(\tau ,\nu \right),E^{\left(i-1\right)}\right) & ={\left(E^{\left(i-1\right)}\right)}^{H}r\left(\tau ,\nu \right)\left(r^{H}\left(\tau ,\nu \right)r\left(\tau ,\nu \right)\right){)}^{-1}r^{H}\left(\tau ,\nu \right)E^{\left(i-1\right)} \end{array}$$
  (21) $$\begin{array}{cc} & =\frac{{\left(r^{H}\left(\tau ,\nu \right)E^{\left(i-1\right)}\right)}^{*}r^{H}\left(\tau ,\nu \right)E^{\left(i-1\right)}}{{∥r\left(\tau ,\nu \right)∥}^{2}}=\frac{|r^{H}\left(\tau ,\nu \right)E^{\left(i-1\right)}{|}^{2}}{{∥r\left(\tau ,\nu \right)∥}^{2}}. \end{array}$$
  In this way, at each iteration, the IPI between the paths is directly canceled, facilitating the new search.
- *Global refinement (line 21):* It is important to notice that this solution is prone to error propagation across all estimates. This may lead to residue vectors with residual peaks that are interpreted as small-amplitude paths by the algorithm. In order to avoid false alarms or multidetections due to residual uncancelled IPI, the P-IPIC algorithm needs a final refinement step in which all the estimates are refined while the residue in monitored. If the residue satisfies the stopping criterion, all remaining unrefined estimates are declared false alarms and, therefore, discarded. Specifically, during the *search phase*, a number of paths equal to $\hat{P}$ is detected. After that, during the *refinement phase* the paths are refined as in the DDIPIC algorithm with the only difference in using the cost function in Equation (19) with a Tikhonov regularization, also known as ridge-regression, and in performing channel gain refinement as in line 15. As discussed above, during this phase some paths may be discarded. Therefore, the refinement procedure provides a number of estimated paths equal to $\hat{\hat{P}}\leq \hat{P}$. The number of paths detected during the *search phase* cannot be determined a priori but it is reasonable to assume that, if the IPI is not too large and the channel is made of a sufficiently high number of propagation paths, $\hat{\hat{P}}\approx \hat{P}$.
- *Regularization (line 15):* Another difference from the original version of the IPI cancellation algorithm is that the ordinary least squares (OLS) estimate of channel gains in Equation (18) is replaced by a regularized least squares (RLS) estimate to make the matrix inversion more stable. This is due to the fact that, due to residual IPI, some paths are detected multiple times, and as a result, the CDDPM matrix may have some almost collinear columns. Therefore, the RLS estimate of the channel gains vector is given by
  (22) $$\hat{g}={\left(R^{H}\left(\hat{\tau },\hat{\nu }\right)R\left(\hat{\tau },\hat{\nu }\right)+N_{0}I\right)}^{-1}R^{H}\left(\hat{\tau },\hat{\nu }\right)y$$
  where I is the identity matrix.

### 3.5. Latency/Complexity Comparison

The key advantage of the proposed approach is that it requires a single final refinement procedure compared to the $\hat{P}-1$ refinements performed by the DDIPIC algorithm. A rough latency comparison of the two approaches is shown in Table 1, where we assumed that the P-IPIC algorithm does not overestimate the number of paths significantly during the first iterative procedure; thus, $\hat{\hat{P}}\approx \hat{P}$. Under this hypothesis, and denoting by $T_{\mathrm{coarse}}$ the average time needed to perform a coarse estimate and with $T_{\mathrm{fine}}$ the average time needed to perform a fine estimate, we can make the following observations.

1. DDIPIC: This performs a coarse and fine estimation for every path in a time equal to $\hat{P}\left(T_{\mathrm{coarse}}+T_{\mathrm{fine}}\right)$. Moreover, from the second iteration to the end, it performs $\hat{P}-1$ refinement procedures of the estimates. Therefore, at each refinement procedure, it performs *i* coarse and fine estimates.
2. P-IPIC: This performs a coarse and fine estimation for every path in a time equal to $\hat{P}\left(T_{\mathrm{coarse}}+T_{\mathrm{fine}}\right)$ during the *search phase*. Moreover, the *refinement phase* performs a second coarse and fine estimate for each path.

**Table 1:** Latency comparison of the two IPIC algorithms.

Algorithm	Latency
DDIPIC [11]	$\left(T_{\mathrm{coarse}}+T_{\mathrm{fine}}\right)\hat{P}+\left(T_{\mathrm{coarse}}+T_{\mathrm{fine}}\right)\sum_{i=2}^{\hat{P}}i=$
	$2\left(T_{\mathrm{coarse}}+T_{\mathrm{fine}}\right)\hat{P}+\left(T_{\mathrm{coarse}}+T_{\mathrm{fine}}\right)\left(\frac{\hat{P}\left(\hat{P}-1\right)}{2}-1\right)$
P-IPIC (Prop.)	$2\left(T_{\mathrm{coarse}}+T_{\mathrm{fine}}\right)\hat{P}$

During the *search phase*, the proposed P-IPIC algorithm runs coarse and fine estimation procedures of channel delays and Dopplers performing maximization of the cost function in Equation (21), while during the *refinement phase,* it maximizes the regularized version of Equation (19). The complexity comparison of the cost function in Equation (19) and the RCF is shown in Table 2. It can be noticed that the first advantage in computing the RCF is that it does not require matrix inversion but only multiplications and additions; instead, the computation of the cost function in Equation (19) does. In order to compare the two cost functions, we can make the following observations.

1. DDIPIC cost function in (19): During the *i*-th iteration, the number of required operations is
  (a) $y^{H}R\left(\tau ,\nu \right)$, $y\in C^{MN\times 1}$, $R\left(\tau ,\nu \right)\in C^{MN\times i}$ requires MNi multiplications and (MN−1)i additions. Then, $R^{H}\left(\tau ,\nu \right)y$ is simply obtained applying the Hermitian operator.
  (b) $R^{H}\left(\tau ,\nu \right)R\left(\tau ,\nu \right)\in C^{i\times i}$ requires $MNi^{2}$ multiplications and $\left(MN-1\right)i^{2}$ additions. After that, matrix inversion has a complexity in the order of $O(i^{3})$.
  (c) $y^{H}R\left(\tau ,\nu \right){\left(R^{H}\left(\tau ,\nu \right)R\left(\tau ,\nu \right)\right)}^{-1}$ requires $i^{2}$ multiplications and i(i−1) additions.
  (d) The matrix multiplication of the 1×i matrix obtained in the previous step by ${\left(y^{H}R\left(\tau ,\nu \right)\right)}^{H}$ requires *i* multiplications and i−1 additions.
2. P-IPIC RCF: during the *i*-th iteration, the number of required operations is
  (a) $r^{H}\left(\tau ,\nu \right)E^{\left(i-1\right)}$,$r\in C^{MN\times 1}$,$E^{\left(i-1\right)}\in C^{MN\times 1}$ requires MN multiplications and MN−1 additions. After that, an additional multiplication is needed to compute the squared module.
  (b) ${∥r\left(\tau ,\nu \right)∥}^{2}$ requires MN multiplications and MN−1 additions.
  (c) One final division is required to compute the ratio between numerator and denominator.

The computation of the RCF is independent of the iteration index *i*, while the computational complexity of the cost function in Equation (19) increases as $O(i^{3})$ due to the inversion of a i×i matrix.

In the end, the search cost of the proposed P-IPIC is identical to that of the DDIPIC, since coarse and fine estimates are performed in the same way apart from the cost function. Hence, as in [11], the search cost depends on the number of points in the integer DD grid during the coarse estimate, or the number of points in the fractional search space $F_{DD}^{\left(s\right)}$ during the fine estimate. In particular, during the coarse estimation phase, both algorithms perform (L+1)(2K+1) cost function maximizations, while during the fine estimation phase, they perform at most $\left(2\lfloor \frac{m_{\tau }}{2}\rfloor +1\right)\left(2\lfloor \frac{n_{\nu }}{2}\rfloor +1\right)$ maximizations.

**Table 2:** Comparison of computational complexity of cost functions.

Cost Function	Equation (19)	RCF
Multiplications	$\left(MN+1\right)\left(i^{2}+i\right)$	2MN+1
Additions	$\left(MN-1\right)\left(i^{2}+i\right)+i^{2}-1$	2(MN−1)
Total number of operations	$2MN\left(i^{2}+i\right)+i^{2}-1+O\left(i^{3}\right)$	4MN

## 4. Simulation Results

In this section, the proposed method is evaluated and compared against the baseline DDIPIC method.

### 4.1. Simulation Parameters

To investigate the performance of the proposed approach, we consider OTFS modulation with M=16 and N=16 with subcarrier spacing of Δf=25kHz and a carrier frequency $f_{c}=5.1\;\mathrm{GHz}$. Thus, delay and Doppler resolutions are $\tau_{\mathrm{res}}=2.5\;\mu s,$ and $\nu_{\mathrm{res}}=1.5625\;\mathrm{kHz}$. As in [11], the parameters for running channel estimation algorithms are $P_{\max}=15,\;s_{\max}=10,\;\epsilon_{\tau }=10^{-10},\;\epsilon_{\nu }=10^{-2},\;m_{\tau }=10,\;n_{\nu }=10$. Finally, as discussed above, the convergence tolerance parameter for the stopping criterion is set according to the noise variance and OTFS frame parameters.

### 4.2. Simulation Scenarios

The high-mobility multipath channel is assumed to have P=4 Rayleigh faded paths with exponential power delay profile (PDP) with a decaying time constant of 10μs and a maximum delay of 10μs. We consider two different case studies corresponding to different scenarios:

1. *Low-IPI Regime*: Propagation delays and Doppler shifts assume fractional values but the paths are far enough to guarantee low IPI. Thus, all paths are separable in the DD domain.
  The delays and Doppler shifts of the paths are $\tau =[0\;\;0.86\tau_{\mathrm{res}}\;\;2.11\tau_{\mathrm{res}}\;\;3.2\tau_{\mathrm{res}}]\;s$, $\nu =[-1.87\nu_{\mathrm{res}}\;\;2.24\nu_{\mathrm{res}}\;\;-3\nu_{\mathrm{res}}\;\;1.3\nu_{\mathrm{res}}]\;\mathrm{Hz}$, respectively.
2. *High-IPI Regime*: Propagation delays and Doppler shifts assume fractional values, and the paths are close enough to create high IPI.
  The propagation delays of the paths are τ=[02.33.157.7]μs. The maximum user equipment (UE) speed is $v_{UE}=190\;\frac{m}{s}\left(684\frac{\mathrm{km}}{h}\right)$, resulting in a maximum Doppler spread of $\nu_{\max}=\frac{v_{UE}}{c}f_{c}=3.23\;\mathrm{kHz}$, where $c=3\cdot 10^{8}\;\frac{m}{s}$ is the speed of light.
  The Doppler shifts of all paths are generated assuming Jakes’ Doppler power spectrum (DPS) using $\nu_{i}=\nu_{\max}cos\left(\theta_{i}\right)$ where $\theta_{i}∼U\left[0,2\pi \right]$.

**Figure 1:**
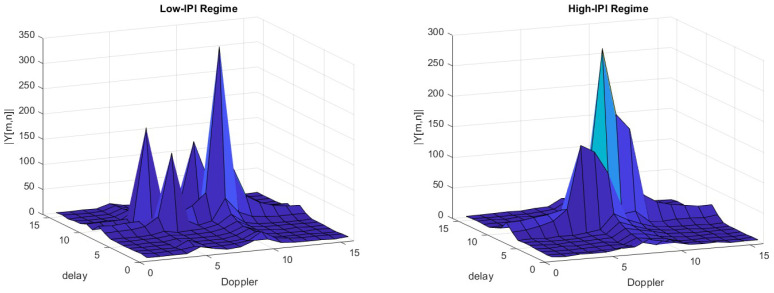
Received DD domain pilot signal.

An example of a pilot frame received in both regimes is provided in Figure 1.

### 4.3. Performance Metrics

We will consider the following performance metrics:

- *Channel estimation:* The accuracy of the channel estimate is measured through the normalized mean square error (NMSE) computed as
  (23) $$\mathrm{NMSE}=E\left[\frac{∥H-\hat{H}{∥}_{F}^{2}}{{∥H∥}_{F}^{2}}\right].$$
- *Channel parameter estimation:* The accuracy of the channel parameter estimates is also considered evaluating the normalized root mean square error (NRMSE) as
  (24) $$\mathrm{NRMSE}\left(\vartheta \right)=\sqrt{\frac{E\left[\sum_{i=1}^{P}{\left|\vartheta_{i}-\hat{\vartheta_{i}}\right|}^{2}\right]}{\sum_{i=1}^{P}{\left|\vartheta_{i}\right|}^{2}}},$$
  where ϑ is a variable that represents delay, Doppler, or channel gain.
- *Model order performance:* The estimation of *P* will be evaluated by the average number of estimated paths and the probability mass function (PMF) of the number of detected paths.
- *Communication:* Communication performance of the proposed approach are evaluated by measuring the bit error rate (BER) as a function of the SNR/bit in order to investigate for the detection robustness when real channel estimation with the proposed variant is performed at the receiver. The SNR/bit is given by $E_{b}/N_{0}$ where $E_{b}$ is the average energy per bit. We consider a quadrature amplitude modulation (QAM) with Q=16 constellation points and $E_{b}=\frac{2(Q-1)}{3log_{2}Q}$. The input–output relation for data frames is the one in Equation (9). At the receiver, a linear minimum mean square error (LMMSE) equalizer provides the estimated symbols as
  (25) $$\hat{x}={\left({\hat{H}}^{H}\hat{H}+\frac{1}{\mathrm{SNR}}I\right)}^{-1}{\hat{H}}^{H}y,$$
  where $\mathrm{SNR}=\frac{MNE_{s}/NT}{M\Delta fN_{0}}=\frac{E_{s}}{N_{0}}$ is the communication signal-to-noise ratio and $E_{s}=E_{b}log_{2}Q$ is the average energy per symbol. After that, a symbol-by-symbol maximum likelihood (ML) detector takes the decision.

### 4.4. Results and Discussion

#### 4.4.1. Channel Estimation Performance

**Figure 2:**
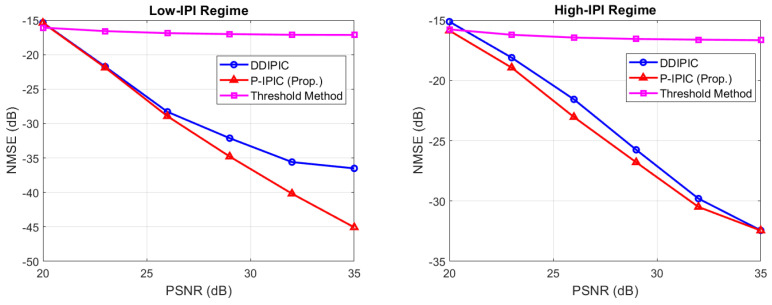
NMSE as a function of pilot SNR.

Figure 2 shows the NMSE as a function of the pilot SNR in both low- and high-IPI regimes. In the low-IPI case at low PSNR, the performance of the proposed variant and the DDIPIC algorithm are comparable, while at higher values of PSNR, the proposed variant outperforms the DDIPIC algorithm, achieving much lower NMSE. On the other hand, in the high-IPI regime, the P-IPIC algorithm achieves an NMSE 1–2 dB lower than the DDIPIC, except for the case of PSNR = 35 dB, where the NSME performances become comparable. We attribute this performance gain to the fact that during the *search phase,* possible paths are detected with the goal of reducing the residue energy, and thus to achieve high accuracy in channel matrix estimate. After that, the good channel estimate obtained in the *search phase* is used to refine the estimates and declare false alarms. The performance of the threshold method in [10] is also presented in both cases as a baseline for comparison, and it can be noticed they are drastically degraded due to the presence of IPI, since it assumes integer DDs.

#### 4.4.2. Channel Parameter Estimation Performance

**Figure 3:**
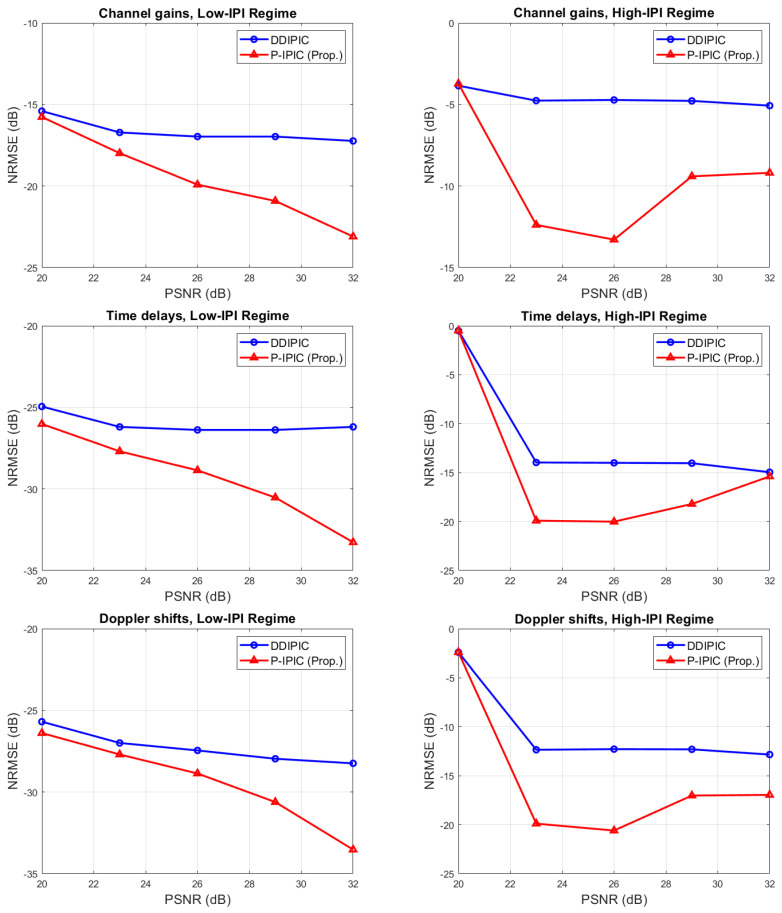
NRMSE of channel parameters as a function of pilot SNR.

Figure 3 shows the NRMSE of channel gains, delays, and Doppler shift as a function of pilot SNR in a channel randomly generated according to channel models 1 and 2. In both low- and high-IPI regimes, the P-IPIC is capable of estimating channel parameters with higher accuracy. It can be noticed that in the high-IPI case, at low PSRN, the NRMSE is high due to the fact that small amplitude paths are missed. On the other side, at high PSNR, the NRMSE for the P-IPIC increases because small errors in the parameter estimates during the *search phase* lead to higher residual IPI that is interpreted as small-amplitude paths, reducing the overall accuracy of the estimates. However, the P-IPIC still outperforms the DDIPIC algorithm for high PSNR.

#### 4.4.3. Number of Detected Paths

**Figure 4:**
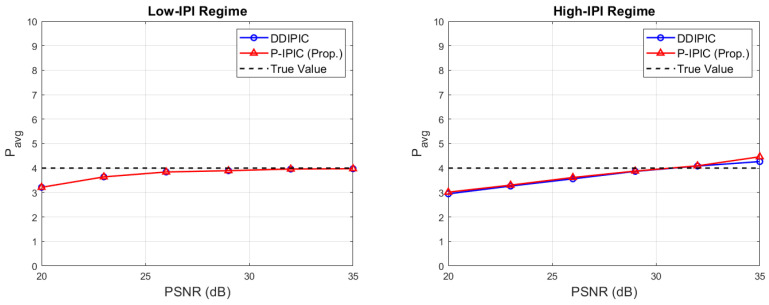
Average number of estimated paths as a function of pilot SNR.

**Figure 5:**
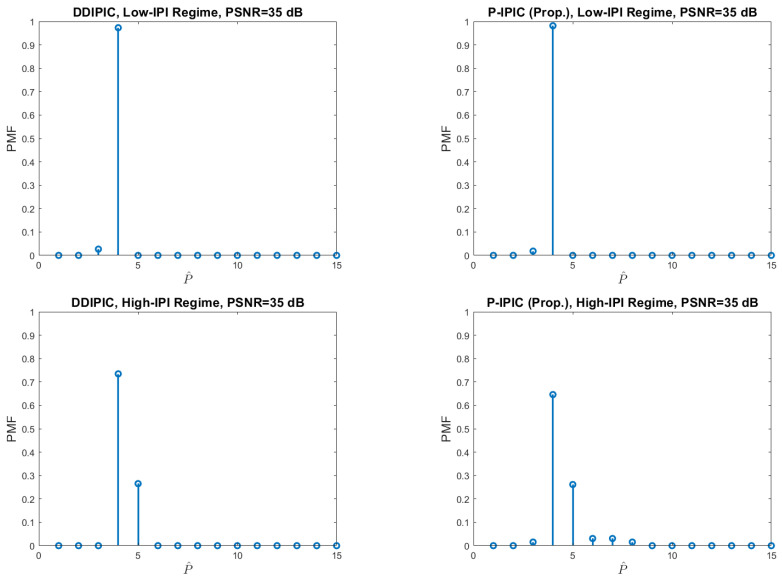
Probability mass function of the number of detected paths.

Figure 4 shows the average number of detected paths as a function of PSNR. In the low-IPI case, performances of both algorithms are comparable. At low PSNR, the average number of detected paths is below the true value (P=4) because small-amplitude paths are missed. At higher PSNR values, both algorithms are able to detect the right number of paths. On the other hand, in the high-IPI case, detection capabilities are still comparable, but at high PSNR values, both algorithms overestimate a bit the number of propagation paths due to high residual interference effects. Figure 5 shows the probability distribution among all the possible values for *P* in the case of PSNR = 35 dB. In the low-IPI regime, both algorithms detect the right number of paths with a probability close to ∼0.98. Thus, the probability of miss detection and the probability of false alarm are close to zero (probability of miss dtection <0.02). In the high-IPI regime, the probability that the algorithm finds the correct number of paths is reduced to ∼0.73 for the DDIPIC and ∼0.65 for the P-IPIC. The probability of miss detection is close to zero in both cases (∼0.015 for the P-IPIC). In the end, the probability of false alarm is ∼0.27 for DDIPIC and ∼0.34 for P-IPIC.

#### 4.4.4. Communication Performance

**Figure 6:**
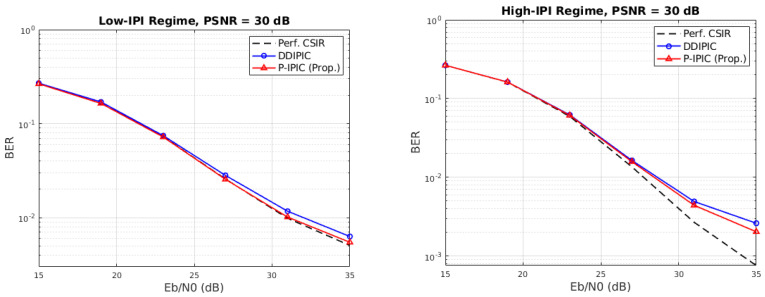
Bit error rate as a function of SNR/bit.

BER performance with the estimated channel (PSNR = 30 dB) and with perfect channel state information at the receiver (CSIR) is provided in Figure 6, showing the BER performance as a function of the SNR/bit in the low- and high-IPI regimes. As expected, the proposed P-IPIC achieves lower bit error rates in both regimes. In addition, BER curves are closer to the ideal curves obtained with perfect channel state information at the receiver.

## 5. Conclusions and Future Works

In this work, we addressed the challenge of low-complexity fractional delay-Doppler (DD) channel estimation in orthogonal time–frequency space (OTFS) systems due to inter-path interference (IPI). A variant of the delay-Doppler inter-path interference cancellation (DDIPIC) algorithm is proposed, which progressively cancels IPI as estimates are obtained, requiring only a final refinement procedure. This approach reduces the latency of the algorithm, with the time difference in latency being almost proportional to the square of the number of estimated paths. Numerical results demonstrate that the proposed algorithm achieves both lower normalized mean square error (NMSE) and bit error rate (BER) compared to other schemes with comparable detection capabilities. The high-IPI scenario is also taken into account, in which the delay and Doppler resolutions are not sufficiently fine, and IPI cancellation becomes tricky.

In future work, the use of deep learning (DL) approaches, as in [24], can be considered to further reduce computational complexity while maintaining good performance and the analysis in presence of interference will be included.

## Data Availability

The original contributions presented in the study are included in the article, further inquiries can be directed to the corresponding authors.
